# Supplementary material for: Widely targeted metabolomics reveals differences in metabolites of Paeonia lactiflora cultivars
Source: PLoS One. 2024 Apr 16;19(4):e0298194. doi: 10.1371/journal.pone.0298194 (PMC11020836; doi:10.1371/journal.pone.0298194)
Supplement: S1 File — (DOCX) [file pone.0298194.s001.docx]

**Table S1.** Sample Numbers

| **Sample tissue** | **Process description** | **Sample name** | **Groups** |
| --- | --- | --- | --- |
| Peony root | No processing | CK1 | Fenyunu |
| Peony root | No processing | CK2 | Fenyunu |
| Peony root | No processing | CK3 | Fenyunu |
| Peony root | No processing | DFG1 | Dafugui |
| Peony root | No processing | DFG2 | Dafugui |
| Peony root | No processing | DFG3 | Dafugui |
| Peony root | No processing | HSML1 | Red charm |
| Peony root | No processing | HSML2 | Red charm |
| Peony root | No processing | HSML3 | Red charm |

**Table S2.** The proanthocyanidin metabolites after multiple differential treatments

| Group | Classification | Compound | up/down  regulated |
| --- | --- | --- | --- |
| CK vs. DFG1 | Proanthocyanidins | 2α,3α-Epoxy-5,7,3',4'-tetrahydroxyflavan-(4β-8-catechin)* | up |
| CK vs. DFG2 | Proanthocyanidins | 2α,3α-Epoxy-5,7,3',4'-tetrahydroxyflavan-(4β-8-epicatechin)* | up |
| CK vs. DFG3 | Proanthocyanidins | Procyanidin A1 | up |
| CK vs. DFG4 | Proanthocyanidins | Procyanidin A2 | up |
| CK vs. DFG5 | Proanthocyanidins | Procyanidin B2 | up |
| CK vs. DFG6 | Proanthocyanidins | Procyanidin B3 | up |
| CK vs. DFG7 | Proanthocyanidins | Procyanidin C1 | up |
| CK vs. DFG8 | Proanthocyanidins | Procyanidin C2 | up |
| CK vs. HSML1 | Proanthocyanidins | 2α,3α-Epoxy-5,7,3',4'-tetrahydroxyflavan-(4β-8-catechin)* | down |
| CK vs. HSML2 | Proanthocyanidins | 2α,3α-Epoxy-5,7,3',4'-tetrahydroxyflavan-(4β-8-epicatechin)* | down |
| CK vs. HSML3 | Proanthocyanidins | Procyanidin A2 | down |
| CK vs. HSML4 | Proanthocyanidins | Procyanidin B1 | down |
| CK vs. HSML5 | Proanthocyanidins | Procyanidin B4 | down |
| CK vs. HSML6 | Proanthocyanidins | Procyanidin B3 | down |
| CK vs. HSML7 | Proanthocyanidins | Procyanidin A6 | down |
| CK vs. HSML8 | Proanthocyanidins | Procyanidin C1 | down |
| CK vs. HSML9 | Proanthocyanidins | Procyanidin C2 | down |
| CK vs. HSML10 | Proanthocyanidins | Galloylprocyanidin C2 | down |
| DFG vs. HSML1 | Proanthocyanidins | Procyanidin B4 | down |
| DFG vs. HSML2 | Proanthocyanidins | Procyanidin C2 | down |
| DFG vs. HSML3 | Proanthocyanidins | Procyanidin C1 | down |
| DFG vs. HSML4 | Proanthocyanidins | Procyanidin B3 | down |
| DFG vs. HSML5 | Proanthocyanidins | Procyanidin A2 | down |
| DFG vs. HSML6 | Proanthocyanidins | Procyanidin A1 | down |
| DFG vs. HSML7 | Proanthocyanidins | Procyanidin B1 | down |
| DFG vs. HSML8 | Proanthocyanidins | Procyanidin A6 | down |
| DFG vs. HSML9 | Proanthocyanidins | 2α,3α-Epoxy-5,7,3',4'-tetrahydroxyflavan-(4β-8-epicatechin)* | down |
| DFG vs. HSML10 | Proanthocyanidins | 2α,3α-Epoxy-5,7,3',4'-tetrahydroxyflavan-(4β-8-catechin)* | down |
| DFG vs. HSML11 | Proanthocyanidins | Galloylprocyanidin C2 | down |
