# Supplementary material for: Widely targeted metabolomics reveals differences in metabolites of Paeonia lactiflora cultivars
Source: PLoS One. 2024 Apr 16;19(4):e0298194. doi: 10.1371/journal.pone.0298194 (PMC11020836; doi:10.1371/journal.pone.0298194)
Supplement: Attachment — Submitted filename: Review Comments oct 23.docx [file pone.0298194.s007.docx]

**Review Report:**

The manuscript entitled **“Widely targeted metabolomics reveals differences in metabolites of *Paeonia lactiflora cultivars*”** seems to be an interesting study in which authors made attempts to contribute the field.

**Note: I reviewed the file named (PONE-D-23-30697) downloaded from journal online portal and all my comments are according to the line numberings of the pdf file “PONE-D-23-30697”**

**Widely targeted metabolomics reveal differences in metabolites of Paeonia lactiflora cultivars.**

**Detailed Comments/Recommendations:**

**Title:**

- Title of the study is quite justifiable.

**Abstract:**

- Lines 17-19: Objective of the study is not clear
- In line 17 and 18 provide full forms of CK, DFG and HSML.
- Line 20-23: Results are mentioned in this portion instead of methodological procedures. Please write down method and procedure here.
- Results are poorly expressed.
- Conclusion in abstract must be comprehensive.

**Key Words:** Authors used 3 out of 4 same words as mentioned in title. The key word should be different from the words used in title so that the reader may explore the study with different more focused words.

**Introduction:**

- Kindly add the introduction (family, genus, distribution, cultivation etc) of *P. lactiflora.*
- In line 63 add on the names of some metabolites of *P. lactiflora.*

**Materials and Methods:**

**Plant Materials:**

- From where plant material was identified? Add on the details like identification or herbarium no etc.

**Plant extraction:**

- Line 80: Kindly mention the centrifuging in gram units instead of rpm.
- Reference is missing for plant extraction method at end of line 83.

**Results & Discussion:**

- Discussion is well written by the authors.

**Conclusion:**

- Satisfactory.

**References:**

- The authors are advised to review all the references for the strict adherence of the uniformity of Journal’s references style.
- Lines 284-285: Reference is incomplete. Add page number.
- Lines 332-334: Correct the reference according to the journal format.

**Concluding Remarks:**

**The paper needs above mentioned corrections along with the English Language proof readings by some language expert. Then after fulfilling the corrections the paper will be acceptable.**
