# Supplementary material for: Widely targeted metabolomics reveals differences in metabolites of Paeonia lactiflora cultivars
Source: PLoS One. 2024 Apr 16;19(4):e0298194. doi: 10.1371/journal.pone.0298194 (PMC11020836; doi:10.1371/journal.pone.0298194)
Supplement: Attachment — Submitted filename: Response to Reviewers second.docx [file pone.0298194.s009.docx]

Response to Reviewers

[PONE-D-23-30697]

Dear Sir/Madam：

Thank you very much for your last letter and advice. This letter is in response to two reviewers questions.

Please note: Blue and red are the contents of this reply.

Major concerns:
1. Pure metabolomics lacks standards. It is recommended to confirm the major predicted components by classical HPLC quantification with the standards.
Reply: (1) High resolution mass spectrometry AB sciex TripleTOF6600 was used for qualitative detection of mixed samples for plant wide target, and then AB sciex6500 QTRAP was used for quantitative determination. Combined with the advantages of non-targeted and targeted metabolomics, high resolution mass spectrometry was used for accurate characterization. Triple quadrupole mass spectrometry with high sensitivity, high specificity and excellent quantitative capability is used as a complementary tool.
(2) The matching parameters in metabolite identification are Q1 precise molecular mass, secondary fragment, retention time, isotope.
(3) Qualitative and quantitative mass spectrometry analysis of metabolites in samples is based on the MetWare database (MWDB) and Multiple Reaction Monitoring (MRM). The identification of metabolites is based on the exact mass of metabolites, MS2 fragments, MS2 fragment isotope distribution and retention time (RT). Through the intelligent secondary spectrum matching method independently developed by MYWAY Company, the secondary spectrum and RT of metabolites in the project samples are intelligentially matched with the secondary spectrum and RT of the company database. The MS and MS2 tolerances are set to 20 ppm and the RT tolerances to 0.2min.
Thank you for clarification. Because the calibration curves are not shown and only relative abundance of ionized molecules can be shown using MS detector, it should be clearly stated in the manuscript. Still, the standards of major identified compounds should be purchased and analysed in line with the data from MS analysis to improve the chemical identification and quantification value of the work.

Reply: The wide target is a relative quantification method mainly used to compare and analyze the relative content changes of the same substance in different samples, without making calibration curves; The broad target has purchased standard products and established a database. The identification method has been marked, please refer to “Supporting Information3” (“ALL_sample _data.xlsx”). As shown below:

（4）The broad target detection method is to build a database of the standard product, and the metabolites without the standard product will be compared with the secondary spectrum in the public database or the literature. The source of substance identification at the time of database construction can be marked.
（5）Wide target quantitative mode: quantitative gold standard MRM mode, characteristic ion quantitative; Q3, RT, DP, CE of each substance will be optimized, and the peak shape will be better. Accuracy: AB sciex6500 QTRAP quantification; In the MRM model, the four-pole first screens the precursor ions (parent ions) of the target substance, and excludes the ions corresponding to other molecular weight substances to preliminarily eliminate the interference. After ionization induced by the collision chamber, the precursor ion breaks to form a lot of fragment ions, and then a characteristic fragment ion is selected through the triple four-pole filtration to eliminate the interference of non-target ions, so that the quantification is more accurate and the repeatability is better. After the metabolic mass spectrometry data of different samples were obtained, the peak area integral was performed for all the mass spectral peaks of substances, and the mass spectral peaks of the same metabolite in different samples were integral corrected (Fraga et al. 2010).

The language is hard to understand. The brief description could be added to the manuscript, thank you!

Reply: The brief description has been added to the manuscript in line 122-126.

Fraga, C.G., et al., Signature-discovery approach for sample matching of a nerve-agent precursor using liquid chromatogd chemometrics. Anal Chem, 2010. 82(10): p. 4165-73.
Wide target methodology literature: Chen, W, Gong, L Guo, Z, et al,A Novel Integrated Method for Large-Scale Detection, Identification, and Quantification of Widely Targeted Metabolites: Application in the Study of Rice Metabolomics. Molecular Plant, 2013, 6(6): 1769-1780.
The relevant literature with high scores has been published：Zhao ML, Ren YJ , Wei W, Yang JM, Zhong QW and Zheng L. Metabolite Analysis of Jerusalem Artichoke(Helianthus tuberosus L.) Seedlings in Response to Polyethylene Glycol-Simulated Drought Stress, Int. Mol. Sci. 2021, 22, 3294.
Please what means high scores?
Reply: The high score means that the published article is for Chinese Academy of Sciences Zone 2, with an impact factor of 5.6. It's a relatively high grade.
2. Quantification of the components was not shown. Also, the identification confirmation based on a mother total ion and two distinct fragments was not shown.
Reply: Material content data, parent ions, and characteristic fragment ions are detailed in “Supporting Information3”: “ALL_sample_data.xlsx”. The data in the numerical table in the ALL_sample_data table uses the scientific counting method and is the relative content of metabolites, without units, by calculating the peak area formed by the characteristic ions of each substance in the detector; among Q1: Molecular weight of parent ions after the material is added by the electrospray ion source; Q3: Characteristic fragment ions; Sample column: Sample relative content (In lines 132-137).
Thank you. In the supporting information table, the data description is not clear, the form of data not easy to read. The precision for four decimals not seen. The explanation of each column missing. What is mix1 etc. It is unclear what Q1 and Q3 means. There should be two characteristics fragments in high resolution in order to match the identity of the compound. What * means. Overall, a minute amount of thousands of compounds with unclear quantity and quality gives low value for reader.

Reply: “Supporting Information2”(ALL_sample_data.xlsx) has changed the previous scientific counting format to a regular format, with Q1 and Q3 having the precision for four decimals.

As shown below:

Q1: Molecular weight of parent ion after the material is added with ions through the electric spray ion source; Q3: Characteristic fragment ions;

Mix sample is a mixed sample prepared by mixing all sample extracts. There are a total of 3 mix samples in this project, mainly used for quality control;

Metabolite Qualitative Analysis: High resolution mass spectrometry AB science TripleTOF6600 was used to qualitatively detect mixed samples for plant wide targeting, and high-resolution mass spectrometry was used for accurate qualitative analysis.

Metabolite identification is based on the precise mass of metabolites, MS2 fragments, isotopic distribution and retention time (RT) of MS2 fragments. Through Maiwei's self-developed intelligent secondary spectrum matching method, the secondary spectrum and RT of metabolites in project samples are intelligently matched with the company's database one by one. The MS and MS2 tolerance are set to 20 ppm, and the RT tolerance is 0.2 min.

Wide target methodology literature: Chen, W, Gong, L Guo, Z, et al,A Novel Integrated Method for Large-Scale Detection, Identification, and Quantification of Widely Targeted Metabolites: Application in the Study of Rice Metabolomics. Molecular Plant, 2013, 6(6): 1769-1780.
3. How did authors confirm the species varieties? Are there official names and genetic
identification available, could authors perform a genetic comparison study?
Reply: All the *P. lactiflora* samples were sourced from "The National Peony and Peony Germplasm Bank" (In September 2020, National Forestry and Grass Leaf Administration Announces List, [http://chinahhxh.com/indexs.html](http://chinahhxh.com/indexs.html) ) at Luoyang Academy of Agriculture and Forestry Sciences. Provided by Gao Kai, Director of the Germplasm Resources Center, and identified by Professor Jin Mingxian from the Plant Classification Teaching and Research Office of Luoyang Normal University, they are classified as three varieties: "Fenyunu" (CK), "Dafugui" (DFG), and "Red Charm" (HSML) with accurate varieties (In lines 83-89).
The genetic confirmation and specimens information may be needed.

Reply: Dear reviewers pro, the *P. lactiflora* samples were sourced from "The National Peony and Peony ", and there is no problem with the identity of the varieties, and the varieties are accurate. All three varieties have been registered in the Germplasm Bank. and identified by Professor Jin.

Among them, "Fenyunu" and "Dafugui", are traditional varieties, formed spontaneously, without a specific number, and their parents are not clear. Red charm is the American Peony Association gold medal variety, and it can be found on the American Peony Association varietal website

(americanpeonysociety.org/cultivars/peony-registry/). And it is listed in Carsten Burkhardt's Web Project Paeonia database (www.paeon.de/) as 039104.

The confirm of these three varieties may also refer to the following published literature.

[1] Gao K, Li YH, Zhu HZ, et al. Differential Expression Analysis of Flavonoids Metabolites in the Roots of *Paeonia lactiflora* Based on Metabolomics. Molecular plant breeding [J/OL]. <https://kns.cnki.net/kcms/detail/46.1068.S.20230505.0853.004.html>.

[2] Li YH, Guo XM, Gao F, et al. Differential Expression on Analysis of Secondary Metabolite in Different Varieties of *Paeonia laxtiflora* Based on Metabolomics. Northern Horticulture,2023(8): 46-55.

[3] Li YM, Gao J, Wang HJ, et al. Anther culture of peony (Paeonia lactiflora) and establishment of its plantlet regeneration system. Plant Physiology Journal 2022, 58 (12): 2379–2385.

4. How did the authors decide on the HPLC condition of up to only 25% B solvent. In this way, most of the nonpolar compounds will not be eluted.
Reply: The gradient is not 25%; From 5% to 95%, gradient elution see line 109-110. We also have project experience in gradient conditions and have published several articles.
The response is not clear, “project experience”, there should be clear methodology in the article.
Reply: Clear methodology has been added to the article in line 110-112.
5. Which part of plant did the authors extract? Sometimes deep freezing may change the tissues rapidly, affecting the constituents
Reply: The tissue site is the root of the *P. lactiflora*. Our method is freeze-dried. vacuum freeze dry technology referred to as freeze-drying technology, refers to the freezing of the drug solution at low temperature, and then sublimation drying in a vacuum environment to remove water, and then analytical drying after the sublimation, remove part of the combined water method. The technology prevents the physical, chemical and biological properties of the dried material to the greatest extent, and the damage to biological tissues and cell bodies is relatively small.
OK, whether the plant material or its extract was freeze-drier should be clearly mentioned.

Reply: The freeze-dried plant material has been clearly mentioned in line 97.
6. How did authors select 70% methanol as the solvent? Why methanol and not ethanol? Was the condition of the sample kept equal to preserve the proanthocyanin contents?
Reply: Methanol has a simpler structure than ethanol, isopropyl alcohol and other small molecules, so it has stronger cell penetration and can dissolve a larger proportion of substances in cells, thus extracting more metabolites and higher metabolite content. Pure water can only extract polar substances in plants, such as glycosides, amino acids and other extremely polar substances, and the extraction efficiency is low. Pure methanol can extract some non-polar substances (such as free fatty acids, phospholipids) and moderate polar compounds (such as flavonoids phenolamines) in plants. 70% methanol water mixed solvent can extract compounds that can cover most of the metabolites in plants, and the extraction efficiency is higher. Therefore, the optimal method was finally determined to be 70% methanol.
Thank you. Please add briefly to the manuscript.
Reply: The briefly content has been added to the manuscript in line 98-100.

7. Please explain aim and methodology of volcano map, Venn plot, KEGG.
Reply:（1）The volcano map is drawn by ggplot2 (V3.3.0) in R language (V3.5.1); The Volcano Plot is mainly used to show the difference in the relative content of metabolites in the two varieties and the statistical significance of the difference (In line 146-148).
(2) The venn diagrams are drawn using the Venn Diagram (V1.6.20) package in R (V3.5.1). The function of the venn diagram is mainly to show the number of common and endemic substances between different comparison groups (In line 149,200-201).
(3) KEGG enrichment: The process of annotating metabolites to the KEGG pathway according to the cpd_ID of differential metabolites, the pvalue of each pathway was calculated by hypergeometric test, and the ratio of the number of differentially expressed metabolites in the corresponding pathway to the total number of metabolites detected and annotated by this pathway was expressed by Rich Factor. (In line 150-154)
Please add the description to the manuscript.
Reply: Thank you very much.

The methodology of volcano map has been added to the manuscript in line 155-157.

The methodology of venn diagrams has been added to the manuscript in line 157-158 and 208-209.

The methodology of KEGG enrichment has been added to the manuscript in line 159-163.
8. How the statistical analysis was performed?
Reply: (1) PCA analysis: PCA used the built-in statistical prcomp function of R software ([www.r-project.org/](http://www.r-project.org/)), and set prcomp function parameter scale=True, indicating that the data was normalized by unit variance scaling (UV).

PCA uses the built-in statistical prcomp function in R software (www.r project. org/), and sets the prcomp function parameter scale=True to normalize the data using unit variance scaling (UV).
(2) Hierarchical cluster analysis: The metabolite content data were normalized (Unit Variance Scaling, UV Scaling), and the heat map was drawn by R software Complex Heatmap package. The accumulation patterns of metabolites in different samples were analyzed by Hierarchical Cluster Analysis (HCA).

Metabolite content data was normalized using Unit Variance Scaling, and a heatmap was drawn using the R software ComplexHetmap package to perform hierarchical cluster analysis (HCA) on the accumulation patterns of metabolites among different samples.
(3) After log2 transformation of the original data, OPLS-DA carries out Mean Centering processing, and then uses the Metabo AnalystR package OPLSR. Anal function in R software for analysis.
OPLS-DA analysis involved performing a log2 transformation on the raw data, followed by mean centering, and then using the OPLSR Anal function in the MetaboAnalystR package of R software for analysis.
Please add to the manuscript. Please note that some of the description is improper and needs rephrasing: e.g.” Anal function in R software for analysis.”
Reply: PCA analysis has been added to the manuscript in line 145-147.
HCA analysis has been added to the manuscript in line 147-149.
OPLS-DA analysis has been added to the manuscript in line 152-154.
9. Supplementary information is not clear.
Reply: The name of the supplementary information has been changed to “S1_raw_images” in “Supporting Information1” and “Table Supplement” in “Supporting Information2”. The metabolite data of all samples were added in “Supporting Information3”, and the analysis data of metabolite differences among different varieties were added in “Supporting Information4”.
